# Climate change and environmental radioactivity: a review of studies on climate conditions in variation on indoor radon concentrations

**DOI:** 10.1007/s10661-025-13889-8

**Published:** 2025-03-21

**Authors:** Phoka C. Rathebe, Khathutshelo Vincent Mphaga, Daniel M. Masekameni

**Affiliations:** 1 Department of Environmental Health, Faculty of Health Sciences, Doornfontein Campus, University of Johannesburg, P.O. Box 524, Johannesburg, 2006 South Africa; 2 Developmental Studies, School of Social Sciences, University of South Africa, Pretoria, 0003 South Africa

**Keywords:** Radon, Climate change, Temperatures, Buildings, Public health

## Abstract

Climate change is increasingly recognized as a critical factor influencing various environmental and public health issues. This paper discusses the link between climate change parameters and elevated indoor radon levels, aiming to highlight the necessity for urgent public health intervention. By examining temperature fluctuations, precipitation patterns, extreme weather events, and geological changes, the paper elucidates how these factors contribute to the variability of indoor radon concentrations. A review of 31 indoor radon studies from different countries revealed substantial variation in indoor radon concentrations. The weighted mean indoor radon concentration was 178 Bq/m^3^, with a standard deviation of 193 Bq/m^3^. The minimum and maximum concentrations measured were 14.3 Bq/m^3^ and 1083 Bq/m^3^, respectively. Drawing from the findings of other scholars, a significant correlation between climate change and increased radon levels in residential areas has been revealed, suggesting potential health risks for occupants. This paper underscores the urgent need for public health strategies and policies to mitigate radon exposure, enhance awareness, and protect vulnerable populations. There is an urgent need for comprehensive measures, including improved building practices, regular radon monitoring, and robust public health campaigns to address the emerging threat posed by climate-induced radon exposure.

## Introduction

Climate change poses a critical global challenge with significant impacts on environmental health and indoor air quality, particularly regarding radon concentrations in residential settings (Celik, 2020; Al Jassim et al., 2018). Radon, a colorless, odorless radioactive gas resulting from uranium decay in soil and rock, is the second leading cause of lung cancer after smoking (Mäkeläinen et al., 2005; Celebi et al., 2015; Environmental Protection Agency, 2021; Kholopo et al., 2024; Mphaga et al., 2024a, 2024b). Understanding the relationship between climate change and radon levels is essential for developing effective public health strategies. Radon accumulates in poorly ventilated areas, infiltrating homes through structural cracks, construction joints, and gaps around service pipes (World Health Organization, 2009; Environmental Protection Agency, 2021). One of the keyways climate change influences radon concentrations is through temperature variations. As global temperatures rise, soil temperatures also increase, potentially enhancing the release of radon gas from the ground. Warm soil temperatures can increase the rate at which radon is transported from the soil into the atmosphere and subsequently into buildings (Appleton, 2007). Seasonal temperature variations also play a significant role; during colder months, homes are often sealed to conserve heat, leading to higher indoor radon concentrations as the gas accumulates indoors. In contrast, during warmer months, enhanced ventilation may effectively lower indoor radon concentrations, although the efficacy of this approach can vary significantly based on specific construction practices and the prevailing regional climate conditions (Field, 2010). The majority of the published research has demonstrated that cool seasons of the year are often associated with elevated indoor radon concentration compared to warm periods (Denman et al., 2007). However, this relationship is not consistent across the globe depending on various factors such as occupants’ ventilation habits. For example, in Arizona and Alabama, elevated indoor radon concentrations were reported during summer, partly because homes are tightly sealed and air-conditioned during summer and often ventilated during cool seasons (Xie et al., 2015).

Changes in precipitation patterns due to climate change can significantly impact soil moisture levels, which in turn affect radon movement (Nunes et al., 2022). Increased rainfall can lead to higher soil moisture, creating a barrier that slows radon migration from the ground into the air (Victor et al., 2019). However, during periods of drying following heavy rains, the soil can release radon more readily (Friedmann, 2017; Mose et al., 2010). Conversely, prolonged drought conditions can dry out the soil, increasing its permeability and enabling radon to more easily migrate through the soil and into buildings. Thus, fluctuating precipitation patterns contribute to the dynamic nature of radon levels in residential environments (Appleton, 2007). In response to climate change, there has been a push toward more energy-efficient buildings with better insulation and reduced air leakage (Brambilla et al., 2020). While these measures are beneficial for reducing energy consumption, they can inadvertently increase indoor radon concentrations. Tighter building envelopes and improved insulation reduce natural ventilation, trapping radon indoors and allowing it to accumulate at higher levels (Environmental Protection Agency, 2021). Ventilation systems designed to conserve energy may not adequately remove radon, necessitating the implementation of specific radon mitigation strategies alongside energy efficiency measures (Appleton, 2007). Extreme weather events, such as storms, floods, and high winds, are becoming more frequent and intense due to climate change (Walsh et al., 2020). These events can alter building pressures and ventilation patterns, which can either increase or decrease radon levels depending on the specific conditions (McGrath et al., 2021). For instance, flooding can saturate the soil and change the pressure dynamics around a building, potentially increasing radon ingress (Friedmann, 2017). Flood-damaged buildings may also have compromised foundations and walls, providing new pathways for radon entry. Storms and high winds can affect indoor air pressure, influencing the movement of radon into and within buildings (Rey et al., 2022).

Climate change–induced geological and soil changes may also affect radon emission patterns (Koike et al., 2015). Thawing permafrost in cold regions can release trapped radon gas from the soil, increasing indoor radon levels in buildings constructed on or near these areas (World Health Organization, 2009). Land use changes, driven by climate adaptation strategies, can disturb the soil and potentially increase radon emissions (Datta et al., 2025). Erosion and other geological changes can alter the natural radon emission patterns, highlighting the need for ongoing monitoring and adaptation of building practices to manage radon risks effectively (Appleton, 2007). Understanding how climate change influences radon concentration in residential houses is essential for developing comprehensive strategies to protect public health (World Health Organization, 2009; Environmental Protection Agency, 2021). As climate change continues to alter environmental conditions, it is crucial to integrate climate science, building engineering, and public health efforts to address the dynamic challenges posed by indoor radon. This manuscript discusses the link between climate change parameters and elevated indoor radon levels to enable an urgent call for public health intervention.

### Indoor radon concentration and climate change

#### Indoor radon concentration (IRC)

Indoor radon concentration (IRC) is affected by a variety of interconnected factors, including geological properties of the site, architectural features of the building, occupants’ habits, and prevailing meteorological conditions (International Atomic Energy Agency, 2019; Spasić et al., 2022; Rey et al., 2024). These variables collectively contribute to the overall level of indoor radon. While previous studies have mainly examined the influence of meteorological factors on IRC **(**Rey et al., 2022), this article explores the significant impact of geological factors, building characteristics, occupants’ behavior, and meteorological conditions on IRC. It is anticipated that radon exposure will rise in the coming years due to the direct and indirect effects of climate change on meteorological, geological, and anthropological factors associated with indoor radon levels **(**Field, 2010; Akinyemiju et al., 2022). The data presented in Tables 1 and 2 demonstrates the substantial geographical variation in indoor radon concentrations (IRCs), influenced by a combination of climatic, geological, and anthropogenic factors. In this article, thirty-one (31) research articles were reviewed in order to explore the complex interplay between various factors and indoor radon concentrations. Table 1 presents a synthesis of studies examining the influence of seasonal temperature variability on indoor radon concentration (IRC) across diverse geographical locations, encompassing variations in climate, geology, and dwelling construction. To determine the regions’ climate, the Köppen-Geiger climate classification **(**Kottek et al., 2006) and information contained in the respective research articles were utilized. In regions characterized by temperate climates, such as Greece (Papaefthymiou et al., 2003), Spain **(**Cortina et al., 2008), Japan **(**Suzuki et al., 2010), Switzerland **(**Kropat et al., 2014), South Korea **(**Park et al., 2018a, 2018b), Czech Republic **(**Senitkova et al., 2019), and Romania **(**Sferle et al., 2020), studies have shown that indoor radon concentrations (IRCs) are higher in autumn and winter due to reduced ventilation as windows and doors are closed for warmth. This limits radon dispersion and can enhance the stack effect (caused by temperature gradients between indoor and outdoor environments), pulling radon from the soil into homes. Similarly, arid and tropical climates, such as Yemen, Saudi Arabia, and India, demonstrated higher IRCs during cooler periods. While ventilation changes due to temperature are a common factor, climate-specific influences also play a role. These trends are linked to fluctuations in soil moisture and atmospheric pressure, impacting radon emanation and transport, with lower moisture in arid regions promoting radon release and the monsoon influencing gas transport in tropical areas (Shaikh et al., 2003; Alghamdi et al., 2014; Duggal et al., 2014).

**Table 1:** Synthesis of seasonal temperature variability influences on indoor radon concentration (IRC) highlighted in some literature

Authors	Year	Country	Sample size	Geological, climate, and dwelling information	Mean concentration of radon in indoor air	Major findings
Papaefthymiou et al	2003	Greece	58	Greece exhibits a Mediterranean climate, underlain by Triassic igneous and Pliocene–Pleistocene sedimentary geology, with building materials incorporating fly ash–enhanced cement	41 Bq/m^3^	Indoor radon levels varied by season, peaking in winter
Shaikh et al	2003	India	8	Mumbai is tropical wet and dry, featuring consistent high temperatures year-round and a pronounced monsoon season with substantial rainfall. The multi-story building was constructed with concrete	41 Bq/m^3^	Elevated indoor radon levels were observed during winter compared to summer season
Khayrat et al	2003	Yemen	241	Yemen has arid coastal (Hodeidah) to temperate highland (Dhamar). Buildings are characterized by ground floor construction, the absence of basements, and small windows	42 Bq/m^3^	Elevated radon concentrations correlated with higher altitudes, attributed to decreased ventilation resulting from lower average temperatures and consequent window closure
Mäkeläinen et al	2005	Finland	305	Finland has a cold climate and airtight building construction, naturally high uranium concentrations in soil, permeable esker geology, and, occasionally, radon-rich well water (Weltner et al., 2002)	104 Bq/m^3^	Radon concentrations displayed variation throughout the day and across different seasons
Cortina et al	2008	Spain	233	Galicia’s geology features ancient, uranium-rich, highly fractured bedrock. This study was conducted in an environment exhibiting seasonal radon variations (suggesting a temperate climate)	253 Bq/m^3^	Indoor radon was higher during cold periods (autumn to winter) than in the hot periods (spring and summer)
Suzuki et al	2010	Japan	3461	Japan has a temperate climate with seasonal variations. Hence, modern Japanese homes are generally characterized by improved airtightness and decreased natural ventilation	14.3 Bq/m^3^	Factors affecting indoor radon included long-term and seasonal climate changes
Abkari et al	2013	Sweden		Sweden has a cold climate and uranium-rich geology, and most of the dwellings were constructed using materials like alum shale–based concrete contributing to dwellings with reduced air exchange (Radiation & Nuclear Safety Authority, 2009)		Higher air exchange rates were linked to reduced indoor radon concentrations. The lowest radon levels were recorded at temperatures ranging from 20 to 22 °C and a relative humidity of 50–60%
Kropat et al	2014	Switzerland	212 000	Switzerland’s geology comprises uranium-rich granitic Alps, karstified carbonate Jura Mountains, and a sedimentary Swiss Plateau overlying molasse, with glacial deposits. Switzerland has a temperate climate	189 Bq/m^3^	Indoor radon levels were found to decrease with higher outdoor temperatures and at higher altitudes
Alghamdi and Aleissa	2014	Saudi Arabia	786	Study area was situated at 630 m above sea level, exhibited a hot arid desert climate, and featured buildings primarily constructed of brick and concrete	24.68 Bq/m^3^	Radon levels were found to be higher during the winter season
Duggal et al	2014	India	50	Has a hot arid climate, characterized geologically by Aravalli and Vindhyan Super Group rocks, frequently overlaid by windblown sand and alluvium. Dwellings predominantly utilized cement, brick, and local materials, resulting in limited ventilation	143.21 Bq/m^3^	Seasonal radon level variations were observed, with higher concentrations in winter and autumn compared to summer and rainy seasons
Xie et al	2015	USA	1	The north-central USA exhibits a humid continental climate with lake-effect influence, while the broader region’s geology is characterized by diverse sedimentary rocks, faulting, and karst topography (Hahn et al., 2023; Xie et al., 2015)	1083 Bq/m^3^	During autumn and winter, indoor radon levels were found to be higher compared to spring and summer
Park et al	2018	South Korea	599	South Korea has a temperate climate	70.8 Bq/m^3^	Cold weather, particularly winter, contributed to higher indoor radon levels compared to warmer summer months
Park et al	2018	South Korea	296	South Korea, with its temperate climate, is characterized by uranium-rich geological formations such as the Okchon belt, granite, and gneiss	117.86 Bq/m^3^	Factors increasing indoor radon were cold weather and wind speed
Senitkova and Kraus	2019	Czech Republic	1	The Czech Republic, characterized by a temperate climate, features structures with concrete foundations	102.9 Bq/m^3^	Cold weather, low pressure, wind, and frozen soil increased indoor radon levels
Sferle et al	2020	Romania	6	Romania, with its temperate continental climate and Precambrian crystalline and sedimentary geology, features dwellings constructed with concrete foundations, brick walls, and insulated, modern windows and roofing	382 Bq/m^3^	The indoor radon levels were influenced by various factors such as temperature, wind speed, wind direction, and indoor-outdoor pressure difference

**Table 2:** Dwelling and occupant characteristics influencing Indoor Radon Concentrations (IRC)

Authors	Year	Sample size	Country	Mean concentration of radon in indoor air	Major findings
Papaefthymiou et al	2003	58	Greece	41 Bq/m^3^	IRC varied by floor, peaking at lower levels and decreasing linearly from underground to the 2nd floor. Basements and central heating systems influenced ground floor radon levels
Shaikh et al	2003	8	India	41 Bq/m^3^	Highest IRCs were observed, at the lowest levels of the dwellings
Khayrat et al	2003	241	Yemen	42 Bq/m^3^	Elevated IRC was associated with reduced ventilation
Mustapha et al	2003	3	Kenya	148.3 Bq/m^3^	Elevated IRCs were detected in groundwater sources and within building basements
Mäkeläinen et al	2005	305	Finland	104 Bq/m^3^	Dwellings typically had the highest IRC, followed by workplaces, public buildings, outdoor environments, and vehicles
Barros-Dios et al	2007	983	Spain	69.5 Bq/m^3^	Factors influencing IRC include the age of the building, interior and exterior building materials, number of stories, distance above the floor, and elevation above sea level
Cortina et al	2008	233	Spain	253 Bq/m^3^	IRC was associated with ventilation habits
Rahman and Anwar	2008	80	Pakistan	40 Bq/m^3^	IRCs were higher in the bedrooms and basements compared to the living rooms and first floors
Suzuki et al	2010	3461	Japan	14.3 Bq/m^3^	IRCs were highest in mid-1980s homes and decreased thereafter. Factors affecting indoor radon included dwelling age, building materials, and dwelling type (particularly air-tight and reduced ventilation buildings)
Abkari et al	2013		Sweden		Ventilation rate was inversely proportional to IRC. Furthermore, indoor temperature and air exchange rate were associated with indoor radon concentrations
Kropat et al	2014	212,000	Switzerland	189 Bq/m^3^	Lower IRCs were observed in apartments compared to individual houses. Other factors influencing radon levels included ventilation rate, dwelling age (higher levels in buildings constructed after 1900 and again after 1970), foundation type (lower levels in buildings with concrete foundations), and floor level
Alghamdi and Aleissa	2014	786	Saudi Arabia	24.68 Bq/m^3^	Homes without proper ventilation systems and central air conditioners showed elevated IRC, while well-ventilated homes with red bricks and water-based air conditioning systems displayed lower concentrations of radon
Duggal et al	2014	50	India	143.21 Bq/m^3^	Well-ventilated dwellings have lower IRC than poorly ventilated ones. The highest indoor radon concentration was found in mud-type dwellings
Xie et al	2015	1	USA	1083 Bq/m^3^	Dwellings with basement, lacking adequate ventilation, exhibited elevated radon concentrations. Indoor radon concentrations positively correlated with outdoor barometric pressure, indoor-outdoor temperature difference, and indoor-outdoor barometric difference
Celebi et al	2015	7293	Turkey	81 Bq/m^3^	Low radon concentration was linked to clay loam soil and the Mediterranean climate. The highest radon concentration is linked to volcanic geology and uranium deposits
Fahiminia et al	2016	123	Iran	95.83 Bq/m^3^	IRC varied between bedrooms and living rooms, with bedrooms showing higher concentrations. Villas had significantly higher radon levels than apartments. High IRC was observed in basements
Collignan et al	2016	3233	France	155 Bq/m^3^	Enhanced airtightness, specifically achieved by decreasing air permeability (thermal retrofit), resulted in higher IRC, particularly in coastal areas compared to inland regions
Najam et al	2017	10	Iraq	79.6 Bq/m^3^	“The indoor radon concentration in all values measured in Wassit province is less than the range (200–300 Bq/m3). The less value for indoor radon concentrations might be due to good ventilation conditions of the houses.”
Park et al	2018	599	South Korea	70.8 Bq/m^3^	Indoor radon levels have been linked with factors such as geographical location, construction year (post-control), and the use of groundwater
Park et al	2018	196	`South Korea	117.86 Bq/m^3^	Indoor radon concentration was associated with ventilation patterns
Kunovska et al	2018	2778	Bulgarian	111 Bq/m^3^	Elevated concentrations are observed in non-smoking rooms compared to those designated for smoking
Senitkova and Kraus	2019	1	Czech Republic	102.9 Bq/m^3^	Lifestyle of dwellers and the ventilation rate influenced radon levels. Highest indoor radon concentrations were observed on the first and second floors
Sferle et al	2020	6	Romania	382 Bq/m^3^	Building tightness and ventilation rate significantly influenced IRC. Furthermore, IRC was influenced by the presence of cracks in the foundation
Nikkilä et al	2020	80,000	Finland	143 Bq/m^3^	Indoor radon concentrations (IRCs) were influenced by building age, construction materials, and soil properties, such as permeability and uranium content. Rock-based materials showed higher residential radon levels than wood, and elevated radon was linked to porous soil
Ptiček Siročić et al	2020	15	Croatia	70.9 Bq/m^3^	IRCs were higher in the basement compared to the ground and first-floor rooms
Yarmoshenko et al	2020	478	Russia	49 Bq/m^3^	Energy-efficient buildings had the highest IRC. Old dwellings had lower IRC than new dwellings
Chen	2021	21,818	Canada	82 Bq/m^3^	The study found that IRC was higher in newer homes, single-detached bungalows, homes with basements, geothermal heating, and private well water. Conversely, lower levels were observed in older homes, multi-story houses, multi-unit buildings, homes with oil or wood heating, and those connected to municipal water systems
Suman et al	2021	81	India	81 Bq/m^3^	The increase in the air exchange rate and the decrease in temperature gradient cause low activity. The concentration of radon increases with a decrease in ventilation rates. The radon levels were found to be higher in the winter season than in the other seasons
Gruber et al	2021	50,000	Australia	109 Bq/m^3^	Significant correlations were observed between radon concentration and geology, building age, and ground coupling
Wang et al	2022	800	Beijing	42 Bq/m^3^	Ground floor dwellings had notably higher radon concentrations than other floors, with no significant difference among the latter. Buildings constructed after 2010 had higher radon levels than those built in the 1980s, 1990s, and 2000s
Kamang et al	2023	2,671,242	USA and the District of Columbia	181.4 Bq/m^3^	Radon levels were the highest from October to February, with no specific month consistently surpassing the others in concentration
				Weighted mean	178 Bq/m^3^
				Standard deviation	193 Bq/m^3^
				Minimum	14.3 Bq/m^3^
				Maximum	1083 Bq/m^3^

Geological factors such as soil type, uranium content, and underlying rock formations played a crucial role (Nikkilä et al., 2020). For instance, porous soils and elevated uranium concentrations correlate with higher indoor radon levels (Barros-Dios et al., 2007; Park et al., 2018a, 2018b). Building characteristics also influenced indoor radon concentrations. Older buildings with less stringent construction standards also exhibited higher radon concentrations (Collignan et al., 2016; Yarmoshenko et al., 2020). Energy-efficient measures, while reducing energy consumption, inadvertently increased indoor radon levels by limiting ventilation (Lee et al., 2012; Yarmoshenko et al., 2020). Human behavior influenced indoor radon concentration through ventilation practices and occupancy patterns (Mäkeläinen et al., 2005; Zhang et al., 2013). Increased ventilation can effectively reduce indoor radon concentrations, while prolonged occupancy in poorly ventilated spaces elevates indoor radon exposure. Indoor radon levels depend on the complex function of multiple interacting factors. While climatic conditions play a pivotal role, geological formations, structural attributes of buildings, and human behavior also significantly affect the spatial and temporal variations in radon exposure levels. The studies summarized in Table 2 revealed a weighted mean concentration of indoor radon of 178 Bq/m^3^, which was significantly higher than the global average of 39 Bq/m^3^ (Tsapalov et al., 2022; Kovler et al., 2023).

#### The influence of temperature on radon emission and infiltration

Temperature variations significantly influence indoor radon levels, which poses considerable health risks. As the second leading cause of lung cancer following tobacco use, radon accumulation can be particularly hazardous when individuals are exposed to elevated concentrations over prolonged durations (Riudavets et al., 2022). This section delves into the impact of seasonal temperature variability, soil temperature, indoor air temperature, and outdoor air temperature fluctuations on radon emission and infiltration into buildings. Understanding the relationship between temperature fluctuations and radon levels is essential for effective radon mitigation strategies.

### Seasonal temperature variability

The results presented in Tables 1 and 2 indicate that the concentrations of radon and its decay products in indoor environments exhibit both temporal fluctuations and seasonal variability. Seasonal variations in indoor radon concentration are influenced by changing weather conditions and environmental factors such as temperature, pressure, humidity, and wind dynamics (Belete et al., 2022; Elmehdi et al., 2024). Notably, seasonal temperature fluctuations significantly impact the behavior of radon gas (Miklyaev et al., 2021). The outdoor temperature fluctuations play a crucial role in determining occupant behavior, such as the need for ventilation in the summer and heating in the winter. These behaviors also have a significant impact on radon dynamics, including the stack effect and ventilation patterns (Rey et al., 2022). The stack effect refers to the movement of air into and out of buildings due to pressure differences caused by temperature gradients (Li et al., 2018). During the colder months, homes are typically sealed to conserve heat, which can lead to higher indoor radon concentrations (Arvela et al., 2014). The reduced ventilation in winter prevents radon from escaping, causing it to accumulate indoors. Studies have shown that indoor radon levels can be significantly higher in winter compared to summer (Miles et al., 2012; Zhang et al., 2013; Health Canada, 2013; Al-Khateeb et al., 2017; Senitkova et al., 2019). For example, in a study conducted in the USA, radon concentrations were found to be 20–40% higher in winter than in summer due to reduced ventilation and increased use of heating systems (Appleton, 2007). Seasonal variation in indoor radon concentrations is well documented (Papaefthymiou et al., 2003; Shaikh et al., 2003; Cortina et al., 2008; Alghamdi et al., 2014; Park et al., 2018a, 2018b).

Colder outdoor temperatures create a pressure difference, drawing radon-laden soil gas upwards. Conversely, in summer, higher outdoor temperatures can reverse the stack effect, limiting radon entry into indoor space (Rey et al., 2022). Temperature changes also influence the pressure dynamics within buildings, affecting radon infiltration. As temperature differences between indoor and outdoor environments increase, the stack effect becomes more pronounced (Dezfuli et al., 2023). In winter, warm indoor air rises and escapes through upper openings, creating negative pressure at the lower levels of the building. This negative pressure can draw radon from the soil into the home through cracks and openings in the foundation (Environmental Protection Agency, 2021). In summer, the reverse can occur, with cooler indoor air creating positive pressure that may inhibit radon entry. However, the effectiveness of natural ventilation depends on the specific design and construction of the building. Buildings with poor insulation or inadequate ventilation systems may not experience significant reductions in radon levels despite seasonal temperature changes (Becker et al., 2014; Pampuri et al., 2018; Cucu et al., 2022). Conversely, during warmer months, homes are more likely to be ventilated naturally through open windows and doors, reducing indoor radon levels (Kovler et al., 2023). However, the impact of seasonal indoor temperature on radon levels can vary depending on the specific building practices and regional climate (Yazzie et al., 2020).

In regions with extreme summer heat, air conditioning and the rising air-tightness of the buildings may result in homes being sealed similarly to winter conditions, potentially maintaining higher radon levels (Friedmann, 2017; Xie et al., 2015). This can result in indoor air that is less diluted by outside air compared to twentieth-century norms (Stanley et al., 2019). Other studies found that radon levels in homes were higher during the summer (Elmehdi et al., 2024; Wilson et al., 1991). These unexpected findings can be explained by the unique climatic conditions of the region. In contrast to regions characterized by colder climates, like northern Europe and North America, the United Arab Emirates (UAE) encounters exceptionally high temperatures and frequent sandstorms during the summer months. As a result, residents are forced to stay indoors with tightly closed windows, leading to reduced ventilation and potentially higher radon levels inside homes. This contradicts the findings in colder regions where increased ventilation during summer usually leads to lower indoor radon concentrations (Elmehdi et al., 2024). For instance, homes in colder climates may experience greater seasonal variations in radon levels compared to those in milder climates. In contrast, a study in Mediterranean climates found less pronounced seasonal variations in radon levels due to more stable temperature conditions and different building practices (Kikaj et al., 2019). The use of heating and cooling systems also affects indoor radon levels. Heating systems that use forced air can increase indoor radon concentrations by drawing radon from the soil into the home. Similarly, central air conditioning systems that create negative pressure in basements can increase radon infiltration (Boardman et al., 2015). To avert devastating and irreversible health problems linked to lung cancer, the EPA recommends the use of radon-resistant construction techniques and the installation of radon mitigation systems, such as sub-slab depressurization, which reduces radon entry by creating a relative negative pressure zone beneath the building foundation, thereby establishing a differential pressure gradient that diverts radon gas away from the building’s interior (Environmental Protection Agency, 2021). These measures are particularly important in regions with significant temperature variations and high radon potential.

The relationship between climate change and indoor air quality, particularly in terms of radon exposure, shows significant regional variability (Field, 2010). With global temperatures currently 1.1 °C above pre-industrial levels (Lee et al., 2024) and projected to rise by 1.5 °C between 2030 and 2035, we can expect more frequent and intense heat events. The phenomenon of global warming, characterized by a marked rise in the average global temperature over the past century, has considerable implications for both environmental and human health. One notable consequence is its potential influence on indoor radon concentrations. Research has established a link between rising global temperatures and elevated levels of radon in the lower atmosphere (Gross, 2002). This trend is expected to affect half a billion people by 2030 (UNICEF, undated), possibly leading to greater dependence on air conditioning and ventilation systems, which could, in turn, impact indoor radon levels. The interplay between air conditioner (AC) usage and radon dynamics is complex and context-dependent. A critical factor to consider is the thawing of permafrost due to increasing temperatures, which accelerates radon release into the atmosphere and buildings, thereby raising baseline exposure levels (Akinyemiju et al., 2024). Furthermore, the widespread use of AC—aimed at mitigating heat stress—can result in reduced air exchange rates in tightly sealed buildings, potentially contributing to increased radon concentrations, especially on upper floors (Akinyemiju et al., 2024). Empirical research on the direct effects of AC on indoor radon levels presents conflicting outcomes. For instance, a study by Kozak et al. (2014) found that operational AC reduced mean radon and attached progeny concentrations. In corroboration, research by the US Environmental Protection Agency (1978) indicated that central heating or AC systems could mitigate indoor radon and its progeny levels by enhancing effective ventilation rates, underscoring the importance of increased ventilation in combating radon accumulation. Conversely, Grzywa-Celińska et al. (2023) provided evidence of significantly higher radon concentrations in air-conditioned rooms in Poland. Their study highlighted the influence of airtight plastic window frames, which resulted in elevated radon levels. Furthermore, Grzywa-Celińska et al. (2023) theorize that AC exacerbates radon transport from the soil into buildings, while the airtight nature of modern windows further decreases natural ventilation, compounding radon accumulation. These divergent findings illustrate the multifaceted nature of radon dynamics in indoor environments, influenced by factors such as building materials, ventilation strategies, and regional soil characteristics. The contrasting conclusions between the studies by Kozak et al. and Grzywa-Celińska underscore the impact of methodological variations and the specific building contexts. It is worth noting that the impact of AC on indoor radon exposure is not uniform. While some studies suggest that AC can facilitate ventilation and ultimately reduce radon levels (United States Environmental Protection Agency 1978; Kozak et al. 2014), others indicate that it may worsen radon accumulation, especially in tightly sealed structures (Akinyemiju et al., 2024; Grzywa-Celińska et al., 2023). This complexity necessitates a detailed understanding of building characteristics, ventilation approaches, and regional climatic variables to effectively manage indoor radon exposure in the face of ongoing climate change.

The evolving climate is projected to extend the growing season and bring about milder weather. Climate change is expected to influence indoor radon concentrations and its decay products through several mechanisms. While precise predictions are complex, it is likely that climate change will extend the duration of spring and autumn periods when windows are frequently opened, especially in northern US regions and among lower-income populations (Nazaroff, 1992). The dynamics of indoor radon concentration are significantly affected by the efficacy of ventilation and airflow patterns. Increased ventilation rates, marked by the effective introduction of outdoor air, promote the dilution and subsequent dispersion of radon gas, thereby reducing indoor accumulation (Abed et al., 2024). In contrast, homes with limited ventilation—often the result of enhanced sealing for thermal efficiency—are more susceptible to elevated radon levels, as the restricted air exchange hinders the dissipation of radon emanating from subsurface sources (Field, 2010). Recent studies have supported this hypothesis, indicating that the traditional assumptions about indoor radon fluctuations, especially the winter peaks, may no longer be universally applicable (Elmehdi et al., 2024). This shift is likely influenced by various factors, including climate change–induced alterations in ventilation patterns and building characteristics (Stanley et al., 2019). The general trend toward less pronounced seasonal variations in indoor radon concentrations implies that the traditional “seasonal correction values” used in epidemiological studies may no longer be accurate in many regions (Carlo et al., 2023).

### Soil temperature

Soil temperature plays a pivotal role in regulating indoor radon levels. Soil temperature also affects radon emission. In cold climates, permafrost soils act as a significant barrier that restricts radon gas migration from the subsurface into inhabited spaces (Zhang et al., 2024). As global temperatures rise, the soil temperature increases, potentially enhancing the release of radon gas from the ground. Warm soil temperatures can increase the diffusion rate of radon, allowing it to move more freely from the soil into the atmosphere and subsequently into buildings (Baltrocchi et al., 2023). Climate change is accelerating the degradation of permafrost, a frozen layer of soil found in the Northern Hemisphere and China (Glover, 2008; Puchkov et al., 2022; Zhang et al., 2024). Current literature indicates that the degradation rate is expected to increase with ongoing global warming, which could significantly elevate the risk of radon migration (Zhang et al., 2024). This phenomenon enhances the potential for radon intrusion into both residential and occupational environments, raising concerns about indoor air quality and associated health risks. This rapid degradation raises critical issues regarding the dynamics of radon, yet empirical studies examining radon behavior in frozen geological formations are surprisingly limited. As a result, the impacts of thaw-induced changes on radon migration pathways remain inadequately characterized. Most current research is largely theoretical and lacks substantial experimental validation (Puchkov et al., 2022).

The presence of uranium-rich rocks in these areas provides a significant source of uranium (U) and its decay product, radium (Ra), leading to elevated levels of radioactive elements in soils and groundwater (Zhang et al., 2024). Permafrost, which acts as a natural barrier to radon migration, has undergone degradation due to climate change, thus heightening the risk of indoor radon exposure (Glover & Blouin, 2022; Akinyemiju et al., 2024; Zhang et al., 2024). Permafrost, in its natural state, effectively lowers indoor radon levels to 4–8 Bq/m3, resulting in an 80–90% reduction from the global average Ra-226 activity of 39 Bq/kg. Simultaneously, it causes a substantial increase in sub-permafrost radon concentrations, reaching 445.8 Bq/m3, a rise of 11.43 times (Glover & Blouin, 2022). A study conducted by Puchkov et al. (2022) established a direct correlation between permafrost thaw and radon flux density (RFD). Seasonal monitoring identified four distinct increases in RFD between March and July, which were temporally linked to rising temperatures and progressive soil thawing. Notably, a significant surge in RFD was observed when the radium source zone thawed, suggesting that deeper thawing events contribute to increased radon emanation, potentially leading to higher indoor radon exposure (Puchkov et al., 2022). These findings were further corroborated by Glover and Blouin (2022), who found that permafrost thaw could result in temporary radon levels exceeding 200 Bq/m^3^ in basements for periods of up to 7 years, peaking at 350 Bq/m^3^ at a depth of 2 m. In contrast, pile-supported structures exhibited minimal radon levels due to effective ventilation, with significant concentrations arising only at permafrost depths exceeding 9 m.

Studies on the interaction between ambient temperature and radon dynamics revealed a complex framework for radon transport in both open-air and subsurface environments (Stoulos et al. 2024; Haquin et al., 2022). Studies by Stoulos (2024) and Haquin et al. (2022) showed that radon exhalation and soil gas concentrations are highly responsive to temperature changes. Stoulos (2024) found that radon entry into a simulated cellar peaked in summer, correlating with outdoor temperatures, while soil gas concentrations were inversely related. Haquin et al. (2022) observed that warming trends drive radon migration from heated soil, while cooling leads to accumulation, highlighting the significant impact of diurnal and seasonal temperature fluctuations on radon behavior outdoors. In contrast, the thermal properties of buildings alter these dynamics. Stoulos (2024) noted the “stack effect,” which enhances radon infiltration due to indoor-outdoor temperature differentials. However, the thermal mass and insulation of structures mitigate rapid temperature changes. This attenuation is supported by Haquin et al. (2022), indicating that while temperature variations do affect radon behavior, the fluctuations’ magnitude is dampened beneath buildings. This results in higher and more rapid radon fluctuations outdoors compared to indoor settings, where the building envelope moderates temperature variations. Additional factors like ventilation, soil moisture, and barometric pressure, as mentioned by Stoulos (2024), further complicate indoor radon dynamics, distinguishing them from their outdoor counterparts. Indoor radon levels are estimated to be approximately 1000 times lower than those found in the underlying soil. Most homes typically draw less than 1% of their indoor air from the soil, primarily relying on outdoor air, which generally has low radon concentrations. However, homes that have low indoor air pressure, poorly sealed foundations, and multiple entry points for soil gas can draw up to 20% of their indoor air from the soil. This can lead to significantly elevated radon levels, even when soil concentrations are moderate (Appleton et al., 2020).

Furthermore, daily air temperature fluctuations can impact radon levels. During the day, the soil surface warms up, increasing the rate at which radon is released (Elmehdi ET AL., 2024). At night, the surface cools, potentially reducing radon release. These diurnal temperature changes can lead to variations in indoor radon concentrations, necessitating continuous monitoring to accurately assess exposure levels (Soldati ET AL., 2022). Studies have demonstrated that higher soil temperatures, as observed during summer months, can lead to a significant increase in soil gas radon concentrations. Arvela et al. (2016) reported a 14% increase in calculated soil gas radon concentration during summer compared to winter values. A rise in the soil temperature from 7 to 25 °C increased the soil air radon concentration by 10–15% (Arvela et al., 2016). Climent et al. (1999) demonstrated an inverse correlation between radon concentration and both air and soil temperatures at a depth of 300 mm, a phenomenon primarily influenced by the dynamics of gas solubility and transport. Higher temperatures reduce the amount of radon that can dissolve in soil moisture. This change causes more radon to exist in its gas form. As a result, gaseous radon moves more easily through the soil and into the air. This process allows radon to escape from the soil, which lowers the radon concentration in the soil itself. In contrast, cooler temperatures elevate radon solubility, allowing more radon to remain dissolved and leading to higher concentrations in the soil. This inverse correlation illustrates the temperature-dependent partitioning of radon between its gaseous and aqueous phases, ultimately influencing its mobility and concentration at the specified depth. Additionally, higher temperatures may stimulate the production of gas carriers, such as carbon dioxide (CO_2_) and water vapor (H_2_O), which can further enhance the transport of radon from deeper soil layers (European Commission et al., 2019). Secondly, temperature variations can have a profound impact on radon accumulation during winter months. The freezing of water in shallower soil layers can create a capping effect, restricting the upward movement of radon. Beneath these frozen layers, the soil is likely to remain unfrozen and relatively permeable, allowing radon to concentrate to elevated levels (European Commission et al., 2019). This phenomenon can contribute to significantly higher indoor radon levels during winter in many regions. It is important to note that the effects of soil temperature and barometric pressure on indoor radon levels can be synergistic (Stoulos, 2024). A combination of increased temperature and decreased barometric pressure can favor the flux of radon from the soil to the atmosphere, resulting in transient disequilibrium and potentially higher indoor radon concentrations. Additionally, the type of soil and geology in a region can influence how temperature changes affect radon emissions. Sandy soils, for example, have higher permeability, allowing radon to move more easily compared to clay soils (Appleton, 2012). Studies conducted in various regions have highlighted these differences (Nguyen et al., 2018; Alonso et al., 2019; Nunes et al., 2022). In a study conducted in Canada, homes built on permeable soils showed significant increases in radon levels during winter due to enhanced soil gas movement caused by the stack effect (Field, 2010).

#### The influence of precipitation patterns on radon emission and infiltration

Numerous weather-related factors influence the ingress of radon into buildings including precipitation and rainfall. All of these factors may potentially be influenced by climate change to differing degrees based on geography. Recent studies have revealed a substantial worldwide rise in surface-specific humidity, primarily linked to human activities (Appleton et al., 2020; Willett et al., 2007). Increased indoor air humidity, often due to intensified precipitation and rising temperatures, can significantly elevate indoor radon concentrations through several mechanisms. Firstly, higher specific humidity indicates greater moisture availability, leading to increased indoor humidity levels. This can disrupt the balance between radon gas and building materials, as damp materials like concrete can release previously adsorbed radon, raising ambient concentrations. Secondly, elevated humidity affects radon transport by influencing air density and ventilation patterns, which may hinder effective radon removal through natural or mechanical systems. Increased dampness can also promote mold growth, further altering airflow and surface interactions (Field, 2010; Janik et al., 2014). Thus, the rise in indoor humidity, driven by climatic factors, can contribute to higher indoor radon levels, posing potential health risks (Field, 2010; Janik et al., 2014).

Precipitation patterns have a significant impact on indoor radon concentration, as they affect both the movement of radon in the soil and its infiltration into buildings (Rey et al., 2022; Nunes et al., 2022). Precipitation directly affects soil moisture levels, which in turn influence the emission and movement of radon gas (Spasić, et al., 2022). For example, when the soil becomes saturated with water after heavy rainfall, the pathways through which radon travels can become blocked, reducing its emission into the atmosphere. This can lead to an accumulation of radon gas in the soil, increasing the potential for it to enter buildings through foundations and other openings (Field, 2010; Nunes et al., 2022). For instance, a study conducted in the USA found that periods of heavy rainfall led to higher indoor radon levels in homes due to the increased soil moisture, which limited radon escape to the atmosphere and forced more radon into the indoor environment (Field, 2010). Another study in the UK reported similar findings, indicating that soil moisture content and precipitation patterns are critical factors in radon emission and indoor concentration (Appleton, 2007).

Precipitation can also influence groundwater levels, which affect radon transport (Chen et al., 2016). Increased precipitation raises the groundwater table, potentially bringing radon closer to the surface where it can enter buildings. This is especially relevant in regions with high groundwater levels, where precipitation fluctuations can cause significant variations in indoor radon concentrations (Nunes et al., 2022; Rey et al., 2022). During heavy rainfall, radon dissolved in groundwater can migrate to the surface and enter indoor air. This effect is compounded in homes using well water that may contain elevated radon levels, released during activities like showering or washing dishes (Friedmann, 2017). Studies indicate that such homes can have considerably higher indoor radon levels, particularly after intense rainfall (Chen, 2021; Mustapha et al., 2002; Nunes et al., 2022; Rey et al., 2022). Buildings with poor foundations, cracks, or other openings are more susceptible to radon infiltration during periods of heavy rainfall. Water infiltration can create pressure differences that drive radon from the soil into the indoor environment. Additionally, the moisture from precipitation can seep into building materials, creating pathways for radon entry (Environmental Protection Agency, 2021). For example, homes with basements or crawl spaces are particularly vulnerable to increased indoor radon levels following heavy precipitation (Mustapha et al., 2002; Yazzie et al., 2020). The accumulated water in these areas can enhance radon entry by increasing soil gas pressure and providing additional routes for radon to seep through foundation cracks and joints (Yazzie et al., 2020). Effective radon mitigation in such buildings often requires addressing moisture control and sealing entry points to reduce radon infiltration (Knapp et al., 2020). In areas with distinct wet and dry seasons, indoor radon concentrations may fluctuate significantly throughout the year. For instance, during the rainy season, increased soil moisture and groundwater levels can lead to higher indoor radon levels, whereas during the dry season, reduced soil moisture may lower radon emissions from the ground (Friedmann, 2017). In humid climates, where precipitation is frequent, the impact on indoor radon levels may be more pronounced compared to arid regions (Li et al., 2018). Furthermore, geological factors such as soil type and permeability can influence how precipitation affects radon movement and accumulation. Sandy soils, for example, may drain quickly after rainfall, reducing the duration of high soil moisture conditions and potentially limiting prolonged radon accumulation (Appleton, 2012).

Groundwater plays a significant role in the migration of radon through soil and rock. The presence of water within the soil can serve as a barrier, effectively slowing down radon’s movement (Nunes et al., 2022). However, declining water tables, often caused by extended dry spells and climate change, notably heighten the risk of indoor radon exposure (Elmehdi et al., 2024). During droughts, soil desiccation causes cracks and enhances soil permeability, allowing radon to move from the subsurface into the atmosphere and indoor environments (Appleton et al., 2020; Elmehdi et al., 2024). As groundwater levels drop, previously submerged uranium-bearing bedrock becomes exposed, leading to increased radon production through radioactive decay (Daniels, 2015). This exposure and soil fracturing create pathways for radon to enter buildings, particularly through foundation cracks. Additionally, reduced soil moisture diminishes the soil’s ability to trap radon, facilitating its diffusion (Appleton et al., 2020). This dynamic mirrors radon buildup during ground freezing or dew formation, where surface barriers temporarily block gas escape. Together, exposed uranium sources and increased soil permeability during water table declines significantly raise potential indoor radon concentrations, highlighting the need for effective radon mitigation strategies.

#### Building practices and energy efficiency

The relationship between building practices, energy efficiency, and elevated indoor radon concentration is complex and multifaceted. As buildings become more energy-efficient, they often become more airtight to reduce energy loss (Kraus et al., 2014). This, in turn, can impact indoor air quality, including the concentration of radon gas accumulation. Modern building practices aimed at improving energy efficiency often involve creating airtight structures (Yarmoshenko et al., 2020). While this reduces energy consumption for heating and cooling, it can also lead to increased indoor radon levels. Airtight buildings limit the exchange of indoor and outdoor air, trapping radon gas that seeps into the building from the ground (Zhang et al., 2013). Radon, a naturally occurring radioactive gas, enters buildings through cracks in the foundation, walls, and floors, as well as through gaps around service pipes and cables (Mphaga et al., 2024a, 2024b). Studies have shown that energy-efficient homes, particularly those built to meet high insulation standards, can have higher indoor radon levels compared to older, less airtight buildings (Collignan et al., 2016; Khayrat et al., 2003; Papaefthymiou et al., 2003; Sferle et al., 2020; Suzuki et al., 2010; Yarmoshenko et al., 2020). For instance, a study in the USA found that homes built with energy-efficient practices had radon levels 20–30% higher than those of conventional homes (Henschel, 1993). Furthermore majority of the consulted studies also reported that newer dwellings were more likely to have elevated indoor radon levels compared to old houses (Barros-Dios et al., 2007; Kropat et al., 2014; Park et al., 2018a, 2018b; Suzuki et al., 2010). For instance, dwellings built in the nineteenth century had lower radon levels compared to dwellings built in the twenty-first century (Kropat et al., 2014). This is because the reduced ventilation in these homes limits the dilution of radon with outdoor air, causing it to accumulate indoors. Hence, ventilation habit was a significant factor that influenced indoor radon levels in the consulted studies, with poorly ventilated dwellings experiencing elevated indoor radon levels (Suzuki et al., 2010; Kropat et al., 2014; Alghamdi et al., 2014; Park et al., 2018a, 2018b; Senitkova et al., 2019). Elevated indoor radon levels were reported often in single detached dwellings compared to apartment buildings and high-rise buildings (Chen, 2021; Fahiminia et al., 2016). Research indicates that single-family homes are usually built with different materials and ventilation systems compared to apartments and flats (Bulut et al., 2024). Older single-family homes may have building materials that naturally release radon, like concrete and gypsum (Grzywa-Celińska et al., 2020). Furthermore, the ventilation systems in single-family homes may be less effective at eliminating radon compared to those in larger buildings.

The majority of the consulted studies showed that indoor radon level decreases as the height of the dwellings increases. This was evident in studies that have shown that radon levels were higher on the lower floors (i.e., basement and first floor) than on the upper floors (Mustapha et al., 2002; Papaefthymiou et al., 2003; Shaikh et al., 2003; Barros-Dios et al., 2007; Rahman et al., 2008; Ptiček Siročić et al., 2020). The elevated indoor radon levels commonly found in basements throughout the reviewed studies can be attributed to various factors. Radon, being denser than air, has a tendency to accumulate in lower-lying areas. When constructing a building, the process of penetrating the soil surface reaches deeper layers where radon concentrations are often higher due to the presence of the radium isotope 226Ra. This construction process can create pathways for radon to enter the building through the foundation and migrate to the basement level (Grzywa-Celińska et al., 2020). The impact of climate change on indoor radon exposure is complex. Studies have demonstrated that basements in regions such as the USA, where radon levels are usually elevated, are rarely used for purposes other than storage (Field et al., 1998; Xie et al., 2015). Even though basements have elevated radon levels, the associated health risk has been considered minimal due to low basement occupancy (Xioe et al., 2015). However, climate change has the potential to disrupt these patterns (Field, 2010). As temperatures increase due to global warming, individuals may seek cooler areas within their homes, such as basements, during hot summer periods. This shift in behavior could elevate the risk of indoor radon exposure. Energy efficiency measures, such as improved insulation and the use of energy-efficient heating and cooling systems, can inadvertently increase radon concentrations. Insulation reduces the rate of air exchange between indoor and outdoor environments, which can enhance the accumulation of radon indoors (Collignan et al., 2016). Additionally, energy-efficient heating systems, such as ground-source heat pumps, can create pressure differentials that draw radon from the soil into the building (Henschel et al., 1993). Ventilation systems designed to improve energy efficiency can also influence indoor radon levels. Mechanical ventilation systems, if not properly designed and maintained, can create negative pressure zones within the building, enhancing radon infiltration from the ground (Zhang et al., 2013). Conversely, well-designed ventilation systems that ensure adequate air exchange can help mitigate radon levels by continuously diluting indoor air with fresh outdoor air.

Building codes and standards play a crucial role in addressing the dual goals of energy efficiency and radon mitigation. In many countries (particularly in Europe and the USA), building codes have been updated to include requirements for radon-resistant construction techniques, especially in areas known to have high radon potential (Yarmoshenko et al., 2020). These techniques include installing radon barriers, sealing cracks and openings, and providing passive or active sub-slab depressurization systems to vent radon safely outside the building (Environmental Protection Agency, 2021). For instance, the US Environmental Protection Agency (EPA) recommends incorporating radon-resistant features in new construction, particularly in high radon potential areas. These measures are designed to prevent radon entry and ensure that any radon that does enter the building is vented outside, thus maintaining indoor air quality while still achieving energy efficiency goals (Environmental Protection Agency, 2021). Similarly, the Canadian National Building Code includes guidelines for radon control in new buildings, emphasizing the importance of integrating radon mitigation with energy efficiency measures (Health Canada., 2013). To effectively balance energy efficiency and radon mitigation, it is essential to adopt a holistic approach that considers both aspects during the building design and construction phases. Integrating radon mitigation measures with energy efficiency strategies can help ensure that buildings remain safe and healthy for occupants. This includes using radon-resistant construction techniques, installing effective ventilation systems, and conducting regular radon testing and maintenance (Collignan et al., 2016). Furthermore, educating builders, architects, and homeowners about the importance of radon mitigation in energy-efficient buildings is crucial. Increased awareness can lead to better implementation of radon-resistant practices and more proactive approaches to managing indoor air quality (Zhang et al., 2013). Governments and regulatory bodies should also consider providing incentives for the adoption of radon mitigation measures in energy-efficient building projects, thereby promoting the integration of both objectives (Health Canada, 2013).

#### Extreme weather events

Extreme weather events, such as heavy rainfall, flooding, and severe storms, can significantly impact indoor radon concentrations. These events can alter soil moisture levels, groundwater flow, and air pressure differentials, all of which can influence radon entry into buildings. Heavy rainfall and flooding can lead to elevated indoor radon levels by increasing soil moisture and altering the flow of groundwater. When the ground becomes saturated with water, radon gas trapped in the soil is displaced and can more easily migrate into buildings through foundation cracks and openings. Flooding can also damage building foundations and increase the permeability of the ground, creating additional pathways for radon to enter indoor environments (Miles et al., 2012). Studies have shown that periods of heavy rainfall are associated with spikes in indoor radon concentrations. For instance, a study conducted in the UK found that radon levels in homes increased significantly following heavy rain events (Miles et al., 2012). Similarly, research in Finland indicated that homes with high radon concentrations experienced even greater increases during and after periods of intense rainfall (Arvela et al., 2014).

These findings highlight the need for homeowners and building managers to be vigilant about radon testing and mitigation, particularly in regions prone to heavy rainfall and flooding. Severe storms and other extreme weather events can cause rapid changes in air pressure and temperature, which can also affect indoor radon levels. Low-pressure systems, common during storms, can create a vacuum effect, drawing radon gas from the soil into buildings more effectively. Additionally, temperature differentials between the indoor and outdoor environments can influence the stack effect, whereby warm indoor air rises and escapes through the upper parts of a building, while radon-laden air is drawn in from the lower levels to replace it (Collignan et al., 2016). Research has demonstrated that barometric pressure changes associated with storms can lead to fluctuations in indoor radon concentrations (Xie et al., 2015). Studies carried out in Canada found that indoor radon levels were higher during periods of low atmospheric pressure (Chen, 2021; Friedmann, 2017). This correlation suggests that monitoring radon levels during and after extreme weather events can help in understanding the dynamics of radon entry and developing appropriate mitigation strategies. Extreme weather events can disturb soil and groundwater systems, further impacting radon levels (Benà, et al., 2022). Landslides triggered by heavy rains or floods can expose new areas of radon-emitting soil, increasing the radon potential in the affected regions (Appleton, 2012). A study in Japan highlighted the relationship between seismic activity and increased radon levels, noting that radon concentrations in groundwater and indoor air rose significantly following an earthquake (Nagano et al., 2011). This example underscores the broader implications of natural disturbances on radon levels and the need for comprehensive radon risk assessments in areas vulnerable to multiple types of extreme events.

#### Geological and soil changes

While the effects of climate change on indoor radon levels are increasingly concerning, the geological and soil characteristics of a region fundamentally determine its radon potential, independent of climate fluctuations. These factors are vital for evaluating baseline radon risk and for understanding how environmental changes may heighten exposure. The geological and soil characteristics of an area play a critical role in determining indoor radon levels. Radon, a naturally occurring radioactive gas, emanates from the decay of uranium found in soil and rocks. The type of bedrock and soil in a given area directly affects the radon potential. Certain geological formations, such as granite, shale, and phosphatic rocks, are known to have higher uranium content, leading to increased radon emissions. Areas with these types of bedrock typically exhibit higher radon levels compared to regions with sedimentary rocks like sandstone and limestone, which generally have lower uranium concentrations (Nagano et al., 2011; Scheib et al., 2013). For example, studies in the USA have shown that homes built on granitic soils tend to have higher indoor radon levels. In Pennsylvania, which has significant granite formations, elevated radon levels have been reported in residential buildings (Scheib et al., 2013). Similarly, regions in the UK with high radon potential, such as Cornwall and Devon, are underlain by granite bedrock (Miles et al., 2012). These findings underscore the importance of geological surveys in predicting radon risks. Soil permeability significantly influences the movement of radon gas from the ground into buildings. Highly permeable soils, such as sandy or gravelly soils, facilitate the migration of radon through soil pores and fissures. Conversely, clayey soils, which have lower permeability, tend to restrict radon movement (Steck et al., 1999). The presence of fissures, faults, and other geological discontinuities can also enhance radon transport by providing pathways for gas flow. Construction activities and soil disturbances can alter the natural radon flux by changing soil structure and permeability. Excavation, grading, and other forms of soil disruption can increase radon emissions by exposing deeper soil layers with higher uranium content (Averla et al., 2013; Yazzie et al., 2020). Additionally, construction activities can create new pathways for radon entry, such as cracks in foundations and utility penetrations (Environmental Protection Agency, 2021). Evidence from various studies indicates that construction activities can lead to elevated radon levels. Studies conducted in the USA and Poland found that homes built on disturbed soils, such as those in newly developed residential areas, showed higher indoor radon concentrations compared to homes on undisturbed soils (Averla et al., 2013; Dutton et al., 2015; Riudavets et al., 2022). Similarly, research in the UK found that radon levels increased in homes located near construction sites, likely due to soil disturbance and changes in local soil permeability (Miles et al., 2012). These findings emphasize the importance of radon testing and mitigation in areas undergoing significant construction activities.

### The influence of climate change on environmental factors that influence indoor radon concentrations

Indoor radon levels are influenced by various environmental factors, including temperature, precipitation, and atmospheric pressure, which are all subject to changes due to climate change. Studies on the impact of climate change on indoor radon levels have employed a variety of methodologies, ranging from empirical data analysis to predictive modeling. For instance, a study carried out in the United Kingdom (UK) analyzed long-term radon measurements from homes to explore seasonal and interannual patterns (Kikaj et al., 2017). The researchers correlated these variations with changes in external temperature and precipitation patterns, using statistical methods to identify significant trends (IPCC, 2014). The studies reviewed consistently indicate that climate change can significantly influence indoor radon levels. Groves-Kirkby et al. (2010) found that indoor radon concentrations in the UK exhibited notable seasonal variations, with higher levels observed during the winter months. This trend was attributed to increased indoor occupancy and reduced ventilation during colder periods, as well as higher radon exhalation rates from the soil due to lower soil moisture content. In contrast, Arvela et al. (2013) adopted a modeling approach, combining data on soil characteristics, building construction, and meteorological conditions to predict changes in indoor radon levels under different climate change scenarios in Finland. This study integrated empirical radon measurements with climate models to estimate future radon exposure risks. Arvela et al. (2013) projected that rising temperatures and changes in precipitation patterns could lead to increased indoor radon levels in Finland. The modeling results suggested that warmer temperatures could enhance radon diffusion from the ground into buildings, while changes in precipitation could affect soil moisture content and, consequently, radon emanation rates.

Another notable study by Dutton et al. (2015) utilized a combination of field measurements and laboratory experiments to investigate the effects of temperature and humidity on radon emanation from soil and building materials. The researchers measured radon exhalation rates under controlled conditions, simulating various climate change scenarios to determine potential impacts on indoor radon levels. Dutton et al. (2015) provided experimental evidence supporting these findings. The study demonstrated that higher temperatures and lower humidity levels could significantly increase radon exhalation from soil and building materials (Dutton et al., 2015). These results imply that climate change–induced shifts in temperature and humidity could exacerbate indoor radon exposure. The comparative analysis of these studies reveals both commonalities and differences in their findings and implications. One common theme is the significant impact of temperature on indoor radon levels. All three studies highlighted that warmer temperatures could lead to higher radon exhalation rates from soil and building materials. This trend is particularly concerning in the context of global warming, which is expected to increase average temperatures worldwide (IPCC, 2014).

Precipitation patterns also emerged as a critical factor influencing indoor radon levels. Both Groves-Kirkby et al. (2010) and Arvela et al. (2013) noted that changes in soil moisture due to varying precipitation could affect radon emanation rates. Dry conditions, often associated with reduced precipitation, were linked to higher radon levels. This finding underscores the importance of considering regional climate changes, such as droughts, in radon risk assessments. However, the studies also highlighted some differences in regional impacts and mitigation strategies. For instance, Groves-Kirkby et al. (2010) emphasized the need for seasonal radon monitoring and ventilation adjustments in the UK, where winter poses a higher radon risk. In contrast, Arvela et al. (2013) suggested that future building regulations in Finland should account for projected climate changes by incorporating radon-resistant construction techniques.

Recent studies have utilized a mix of empirical data collection, predictive modeling, and experimental approaches to understand the relationship between climate change and indoor radon levels. For instance, Kotrappa et al. (2018) conducted a longitudinal study measuring radon levels in homes across different seasons to observe variations linked to temperature and humidity changes. The findings from these studies consistently highlight the significant impact of temperature and precipitation on indoor radon levels. Kotrappa et al. (2018) found that radon levels in homes were higher during winter months, corroborating earlier findings by Groves-Kirkby et al. (2010). This increase was attributed to lower ventilation rates and higher radon exhalation from drier soils. The study used continuous radon monitors to capture detailed data, which were then correlated with local meteorological conditions. Similarly, Bradley et al. (2017) employed a modeling approach to predict future indoor radon concentrations under various climate change scenarios. The model incorporated data on soil radon concentrations, building characteristics, and projected changes in temperature and precipitation from climate models. This study aimed to forecast potential increases in radon levels and identify regions at higher risk due to climate change. Bradley et al. (2017) projected that future climate change could lead to increased indoor radon levels in certain regions, particularly those expected to experience warmer and drier conditions. The model indicated that areas with high baseline radon levels could see significant increases, posing a greater health risk. An experimental study by Lin et al. (2020) explored how soil moisture and temperature variations influence radon exhalation rates. The researchers simulated different climate conditions in a controlled laboratory setting, measuring radon exhalation from various soil samples under varying temperatures and moisture levels. This approach allowed for a detailed analysis of how specific climate factors directly affect radon emanation (Lin et al., 2020). Lin et al. (2020) provided experimental evidence that supports these projections. Their study demonstrated that higher temperatures and reduced soil moisture significantly increased radon exhalation rates. These findings suggest that climate change could exacerbate radon exposure, especially in regions where soil drying and warming are prevalent (Lin et al., 2020).

Comparing these studies with earlier research reveals a consistent trend: climate change is likely to increase indoor radon levels due to higher temperatures and altered precipitation patterns. The studies by Kotrappa et al. (2018) and Groves-Kirkby et al. (2010) both observed seasonal variations in radon levels, with winter months showing higher concentrations. This seasonal effect is linked to reduced ventilation and higher indoor occupancy during colder periods. The predictive modeling by Bradley et al. (2017) and Arvela et al. (2013) offers insights into future risks, highlighting regions that may experience significant increases in radon levels due to climate change. Both studies emphasize the importance of regional climate projections in radon risk assessments and the need for adaptive building practices. Lin et al. (2020) and Dutton et al. (2015) provide experimental validation of these projections, demonstrating that specific climate conditions, such as higher temperatures and lower soil moisture, directly influence radon exhalation. These findings underscore the need for ongoing monitoring and adaptation in building practices to mitigate radon risks.

A study by Brown et al. (2019) used a combination of empirical data collection and modeling to assess the impact of temperature and humidity on radon levels in residential buildings. By analyzing radon measurements from homes in different climatic regions, the study correlated these measurements with local meteorological data to understand the effect of seasonal and long-term climate changes on radon concentrations (Brown et al., 2019). Another approach by Smith et al. (2020) involved using radon detection sensors combined with climate models to predict future radon levels under various climate scenarios. This study aimed to project how expected changes in temperature and precipitation might affect radon levels in different regions, providing valuable insights into potential future risks (Smith et al., 2020). Additionally, Williams et al. (2021) conducted a field study involving radon measurements in homes subjected to simulated extreme weather conditions, such as prolonged droughts and heatwaves. This study aimed to understand how such conditions might influence radon entry and accumulation in indoor environments. The findings from these studies reveal consistent patterns indicating that climate change factors significantly influence indoor radon levels (Smith etr al, 2020; Williams et al., 2021). Brown et al. (2019) found that increased temperatures were associated with higher indoor radon concentrations, particularly in poorly ventilated homes. This observation aligns with earlier studies by Finkelstein et al. (2016), which also reported elevated radon levels during colder months when ventilation rates are typically reduced. Smith et al. (2020) projected that future climate scenarios involving higher temperatures and altered precipitation patterns could lead to increased indoor radon levels, particularly in regions that are already prone to high radon concentrations (Williams et al., 2021). This study’s projections are consistent with the findings of Clark and Zhang (2018), who highlighted that warming climates might exacerbate radon risks due to changes in soil moisture and ventilation. Williams et al. (2021) found that extreme weather conditions, such as prolonged heatwaves, significantly increased radon concentrations in homes. Their findings were supported by Goh et al. (2019), who reported that extreme weather events could disrupt radon mitigation systems and increase radon entry into buildings.

Earlier studies, such as Young et al. (2012), primarily focused on seasonal variations in radon levels, often overlooking the potential long-term impacts of climate change. The recent studies build on this foundation by incorporating climate models and projections to assess future risks. Brown et al. (2019) and Finkelstein et al. (2016) have contributed to a more nuanced understanding of how temperature affects radon levels, reinforcing the need for adequate ventilation in homes, particularly during colder months. Meanwhile, Smith et al. (2020) and Clark and Zhang (2018) emphasize the importance of considering long-term climate projections in radon risk assessments, suggesting that future research should focus on integrating these projections into building codes and public health strategies. Williams et al. (2021) and Goh et al. (2019) add another layer of complexity by highlighting how extreme weather events can disrupt radon mitigation efforts. These studies underscore the need for adaptive building practices and improved radon mitigation technologies to address the challenges posed by climate change. A substantial body of research links temperature changes with indoor radon concentrations. Bulut et al. (2024), through their review, highlight that rising temperatures influence radon levels by modifying soil radon flux and indoor ventilation. This conclusion aligns with Park et al. (2023), which experimentally demonstrated that higher indoor temperatures could increase radon concentrations. Both studies indicate that warmer temperatures enhance radon entry into homes. Vardoulakis et al. (2015) also reinforced these findings, showing that projected temperature increases could exacerbate radon exposure, particularly in poorly ventilated homes.

## Conclusions

Indoor radon concentration (IRC) represents a complex phenomenon shaped by a convergence of geological, meteorological, architectural, and human factors. Seasonal temperature fluctuations, particularly diminished ventilation during colder months, significantly increase IRCs, a trend observed across various climatic regions. Additionally, climate change—alongside its associated temperature anomalies and changing precipitation patterns—intensifies these issues, potentially leading to greater reliance on air conditioning and tighter building envelopes, which can inadvertently heighten radon accumulation. The thawing of permafrost, driven by rising global temperatures, further aggravates the situation by releasing previously trapped radon into both the atmosphere and buildings. Furthermore, soil characteristics, including permeability and uranium content, are critical in the process of radon emanation and transport, while certain building practices aimed at enhancing energy efficiency can unintentionally create environments that facilitate radon buildup. This intricate interplay of factors highlights the urgent need for comprehensive radon mitigation strategies, such as improved ventilation, radon-resistant construction techniques, and ongoing monitoring, to protect public health in light of changing climatic conditions. The relationship between extreme weather events and elevated indoor radon concentrations has significant implications for radon mitigation and public health policy. Building codes and construction practices should account for the increased risk of radon entry during and after extreme weather events. For instance, incorporating radon-resistant construction techniques, such as improved sealing of foundations and the installation of radon mitigation systems, can help reduce the risk of elevated radon levels during such events.

## Data Availability

All data associated with this manuscript is available upon reasonable request from the corresponding author. No datasets were generated or analysed during the current study.
